# Regulation of Gene Expression in Cancer—An Overview

**DOI:** 10.3390/cells11244058

**Published:** 2022-12-15

**Authors:** Tanguy Ferlier, Cédric Coulouarn

**Affiliations:** Centre de Lutte Contre le Cancer Eugène Marquis, Inserm, University of Rennes 1, UMR_S 1242, OSS (Oncogenesis Stress Signaling), 35042 Rennes, France

Regulation of gene expression takes a central place in normal cells to maintain tissue homeostasis but also in cancer cells to respond to intra- and extra-cellular stimuli, such as therapeutic drugs. Cancer cells integrate these stimuli and activate specific signaling and/or metabolic pathways to facilitate their own survival and to promote tumor progression. One of the key steps in this process is the activation or repression of specific subsets of genes. Thus, gene expression is tightly regulated to initiate a coordinated transcriptional programming that controls fundamental cellular processes associated with cancer cell hallmarks (e.g., proliferation, apoptosis, differentiation, metabolism, migration, and invasion).

This Special Issue of *Cells* aims at providing an overview of the multifaceted aspects of the regulation of gene expression occurring in cancer cells. It is based on review and research articles reporting emerging experimental evidence of gene expression regulation at epigenetic, transcriptional, and post-transcriptional levels.

Omics technologies led to a deep characterization of gene expression signatures specific of the tumor ecosystem, including tumor cells and their environment. As reviewed by Qian et al., these signatures represent a valuable resource to identify clinically relevant tumor subgroups, which was until now mainly based on the anatomical extent of the disease [1]. Thus, prognostic gene expression signatures have been reported in different cancer subtypes, although only a few of them have reached the stage of clinical implementation so far [1]. New integrative tools including multi-omics analysis could push the field forward and improve the clinical outcomes [1].

Functional analysis of cancer-associated signatures could also lead to better understand the phenomena of tolerance and resistance of cancer cells to targeted therapy. As reviewed by Swayden et al., whereas the role of genetic mutations in drug resistance is well-described, recent studies show an important role also for non-genetic mechanisms. Studying these mechanisms will allow the development of new therapeutic strategies to prevent an adaptive mechanism of cancer cells to resist targeted therapy, and thus improve the management of patients with cancer [2]. As part of these non-genetic mechanisms, chromatin remodeling contributes to regulate gene expression in cancer cells. The SWI/SNF complex is an important multi-protein complex for chromatin remodeling. This complex is composed of a dozen proteins including ARID2, whose mutations have been identified in many cancers, including hepatocellular carcinoma (HCC). The precise mechanisms by which the SWI/SNF complex regulates gene expression are diverse and poorly understood. However, this complex appears to provide chromatin rearrangement, which is considered nucleosome ejection and/or sliding. Nucleosome movement facilitates access to chromatin, allowing specific genes to be activated or repressed. SWI/SNF can expose binding sites for transcriptional activators or repressors at gene promoters or enhancers [3]. As reviewed by Loesch et al., the loss of ARID2 in HCC (by mutational inactivation or downregulation by miRNAs), would lead to pro-tumor (e.g., decreased DNA damage response and increased proliferation) and pro-metastatic (epithelial-mesenchymal transition) events [4]. The loss of ARID1A, another subunit of the chromatin remodeling complex, has also been highlighted in HCC. Indeed, *ARID1A* is frequently mutated and ARID1A inactivation is associated with a poor prognosis in patients with HCC. In this Special Issue, Yim et al. provide experimental evidence that the loss of ARID1A in HCC is associated with low immune activity and inactivation of tumor suppressor genes such as *TP53* disrupting TP53-regulated genes such as *CDKN1A* and *SMAD3*, leading to HCC [5]. Accordingly, a low-ARID1A HCC subtype associated with poor clinical outcomes has been identified, suggesting that ARID1A could represent a prognostic biomarker in HCC patients [5].

Histone acetylation is another mechanism involved in chromatin remodeling and subsequently regulating gene expression positively. In this Special Issue, Amin et al. reports that heparanase, an endoglycosidase acting predominantly extracellularly, can be located in the nucleus, where it stimulates histone acetylation, thus enhancing chromatin opening and upregulation of genes that promote an aggressive tumor phenotype. In addition, the authors report that heparanase can decrease the activity of the well-known tumor suppressor PTEN involved notably in the regulation of cell cycle by preventing uncontrolled cell division. Alteration of histone acetylation may therefore play an important role in tumor progression [6].

DNA methylation is an important epigenetic modification regulating gene expression and changes in DNA methylation have been associated with tumor onset and progression. Controlled by methyltransferases, methylation of cytosines modifies the chromatin architecture. It results in compacting nucleosomes and thus preventing the access of DNA to transcription factors. Thus, DNA methylation at the gene promoter regions is usually associated with transcriptional repression. Here, Ummarino et al. report that a nutritional intake of Nicotinamide adenine dinucleotide (NAD), an essential nutrient acting as a coenzyme involved in cellular redox reactions, impacts on the human epigenome. The authors demonstrate that impairment of DNA methyltransferase DNMT1 enzymatic activity by NAD-promoted ADP-ribosylation leads to demethylation and transcriptional activation of the *CEBPA*, a tumor suppressor gene. These findings provide a nutritional approach to the prevention and management of cancers with altered DNA methylation that are likely related to decreased NAD levels [7].

Chromatin remodeling and epigenetic modifications impact the expression of protein-coding mRNAs but also of non-coding RNAs, including microRNAs (miRNAs). Here, Paczkowska et al. highlight miRNAs epigenetically silenced by DNA hypermethylation in Classical Hodgkin Lymphoma (cHL). One of the silenced miRNAs is miR-148a, whose silencing result in the altered control of several target genes, such as *HOMER1* and *IL6*, both of which are involved in the pathogenesis of cHL [8].

The activity of cell signaling pathways is also tightly regulated in cancer by changes in gene expression. Bévant et al. reported an interesting epigenetic control of the well-known functional duality of the transforming growth factor beta (TGF-beta) pathway in liver cancer (i.e., exhibiting cytostatic properties at an early tumor stage but pro-metastatic at later stages). Notably, treatment with decitabine, a demethylating agent used in the clinic for the treatment of several cancers, induces a shift in TGF-beta towards its pro-metastatic action [9]. The authors conclude that epidrugs should be carefully evaluated for the treatment of HCC, as they may activate tumor promoting pathways. Another study by Kim et al. reported a silence of Hippo pathway signature consistently associated with poor prognosis in glioblastoma [10]. The Hippo pathway is known to play an essential role in the regulation of tissue homeostasis. Furthermore, a growing number of reports have suggested that the Hippo pathway contributes to cancer development and progression. This pathway can inhibit the oncogenic transcriptional activators YAP1 and TAZ by phosphorylation.

Therapeutic nucleic acids such as antisense DNA oligonucleotides, or even short interfering RNAs (siRNAs), are useful nucleic acids for regulating gene expression. In this Special Issue, by using EGFR as a paradigm, Gudanis et al. presented a novel strategy for inhibiting translation based on the antisense-controlled formation of an RNA quadruplex–duplex hybrid between a G-rich RNA antisense oligoribonucleotide (Q-ASO) and specific mRNA, comprising two distant G-tracts [11]. The study offers novel insights into the potential application of Q-ASOs for the detection and/or alteration of processes through RNA:RNA quadruplex–duplex formation in cellular systems [11].
